# Homogenous subpopulation of human mesenchymal stem cells and their extracellular vesicles restore function of endometrium in an experimental rat model of Asherman syndrome

**DOI:** 10.1186/s13287-023-03279-7

**Published:** 2023-04-03

**Authors:** Nahid Mansouri-Kivaj, Abdoreza Nazari, Fereshteh Esfandiari, Faezeh Shekari, Marefat Ghaffari, Mohammad Pakzad, Hossein Baharvand

**Affiliations:** 1 Department of Biology and Anatomical Sciences, School of Medicine, Shahid Beheshti University of Medical Sciences, Tehran, Iran; 2 Department of Stem Cells and Developmental Biology, Cell Science Research Center, Royan Institute for Stem Cell Biology and Technology, ACECR, Tehran, Iran; 3 Advanced Therapy Medicinal Product Technology Development Center (ATMP-TDC), Cell Science Research Center, Royan Institute for Stem Cell Biology and Technology, ACECR, Tehran, Iran; 4 Department of Developmental Biology, School of Basic Sciences and Advanced Technologies in Biology, University of Science and Culture, Tehran, Iran

**Keywords:** Asherman syndrome, Stem cell therapy, Clonal mesenchymal stem cells, Extracellular vesicles, Subpopulation

## Abstract

**Background:**

Asherman syndrome (AS), or intrauterine adhesions, is a main cause of infertility in reproductive age women after endometrial injury. Mesenchymal stem cells (MSCs) and their extracellular vesicles (EVs) are promising candidates for therapies that repair damaged endometria. However, concerns about their efficacy are attributed to heterogeneity of the cell populations and EVs. A homogenous population of MSCs and effective EV subpopulation are needed to develop potentially promising therapeutic options in regenerative medicine.

**Methods:**

AS model was induced by mechanical injury in adult rat uteri. Then, the animals were treated immediately with homogeneous population of human bone marrow-derived clonal MSCs (cMSCs), heterogenous parental MSCs (hMSCs), or cMSCs-derived EV subpopulations (EV20K and EV110K). The animals were sacrificed two weeks post-treatment and uterine horns were collected. The sections were taken, and hematoxylin–eosin was used to examine the repair of endometrial structure. Fibrosis was measured by Masson’s trichrome staining and *α*-SMA and cell proliferation by Ki67 immunostaining. The function of the uteri was explored by the result of mating trial test. Expression changes of TNF*α*, IL-10, VEGF, and LIF were assayed by ELISA.

**Results:**

Histological analysis indicated fewer glands, thinner endometria, increased fibrotic areas, and decreased proliferation of epithelial and stroma of the uteri in the treated compared with intact and sham-operated animals. However, these parameters improved after transplantation of both types of cMSCs and hMSCs and/or both cryopreserved EVs subpopulations. The cMSCs demonstrated more successful implantation of the embryos in comparison with hMSCs. The tracing of the transplanted cMSCs and EVs showed that they migrated and localized in the uteri. Protein expression analysis results demonstrated downregulation of proinflammatory factor TNF*α* and upregulation of anti-inflammatory cytokine IL-10, and endometrial receptivity cytokines VEGF and LIF in cMSC- and EV20K-treated animals.

**Conclusion:**

Transplantation of MSCs and EVs contributed to endometrial repair and restoration of reproductive function, likely by inhibition of excessive fibrosis and inflammation, enhancement of endometrial cell proliferation, and regulation of molecular markers related to endometrial receptivity. Compared to classical hMSCs, cMSCs were more efficient than hMSCs in restoration of reproductive function. Moreover, EV20K is more cost-effective and feasible for prevention of AS in comparison with conventional EVs (EV110K).

**Supplementary Information:**

The online version contains supplementary material available at 10.1186/s13287-023-03279-7.

## Introduction

Asherman’s syndrome (AS) affects 2% to 22% of infertile women. Such discrepancy in AS prevalence may related to its subjective diagnosis by clinicians and various social rules about abortion in various countries [1]. It is distinguished by endometrial fibrosis and attachment between endometrial walls due to destruction in the endometrium by repeated or aggressive curettages and/or endometritis [1, 2]. Disease symptoms include incomplete to complete obliteration of the uterine cavity, menstrual abnormalities, secondary infertility, recurrent pregnancy loss and/or abnormal placentation, and placenta previa in conjunction with placenta accreta [1, 2].

Current treatments or preventions for AS rely on reconstruction of the uterine cavity architecture to recover its normal function to increase the chances for a successful pregnancy. Hysteroscopic adhesiolysis followed by hormone therapy or anti-adhesive barriers such as biocompatible hydrogels are routine clinical approaches for AS (for review see [3]). The main limitation of current therapies is recurrence of adhesions in 30% of mild to moderate cases [4] and in 62.5% of severe cases [5–7]. Moreover, uterine perforation, infection, or early miscarriages can occur after these procedures [8], and research is ongoing to develop new therapies.

Human mesenchymal stem cells (MSCs) are one of the therapeutic candidates for AS [9, 10]. The current MSC isolation procedures result in harvests of heterogeneous cell populations that display various phenotypes and characteristics, according to the tissue source, donor, isolation technique, culture protocols, culture media, and passage number [11]. The heterogeneity of this classical MSCs isolation method presents a challenge [12] due to the variations in response and outcome [11]. The subfractionation culturing method is a simple, efficient, and reproducible isolation technique that can be used to establish a homogeneous MSC population. In this technique, single colony forming units (CFUs) are manufactured to produce clonal MSCs (cMSCs) [13–15], which could overcome the challenges faced by the classical approach for isolation of MSCs [16].

MSCs have been shown to improve endometrial function through secreting anti-inflammatory cytokines and growth factors, either directly [17, 18] or through extracellular vesicles (EVs) [19–21] which lead to tissue repair. However, stem cell therapy might raise some concerns such as difficulties with transportation, storage and commercialization, rejection routes, and safety issues due to a lack of proper monitoring tests [22–24].

In contrast, tremendous evidence supports the role for extracellular vesicles (EVs) as new communication paradigms (EV-crine) that can transfer biological information between cells [25–27] in regenerative medicine. EVs are secreted by all cell types under both physiological and pathological situations [28] and convey different macromolecules, including proteins, nucleic acids, and lipids [29]. EVs can be taken up by the target cells and release their cargo inside these cells or they may interact with ligands on the surfaces of the target cells and activate intracellular signaling pathways [30, 31]. EV term is generic term for various types of vesicles secreted by cells, including “exosomes”, “apoptotic bodies” or “microvesicles” [32] and is strongly recommended by the International Society for Extracellular Vesicles (ISEV) to be used [33, 34]. Each cell secrets many types of EVs that differ in terms of content; therefore, they may have differences in physicochemical properties such as density and size [35]. Different types of EVs or EV subpopulations that are isolated by different isolation techniques have different morphologies [36, 37] and functions [38–40]. Specifically, MSCs-EVs are widely used to repair injured tissues, which has been demonstrated in animal models [41, 42] and clinical trials [43–45]. In contrast to MSCs, EVs do not proliferate, and their storage and transfer is easier than viable cells, dosing is not limited by microvascular plugging or loss of viability, and they have high immune tolerability [46, 47]. Moreover, they cryopreserved simply in buffer for a long time. Such unbeatable characteristics of EVs facilitate translational use of EVs as an “off-the-shelf” product and would permit extensive and repeated product testing prior to clinical use.

Although EVs are interesting and effective therapeutic tools, rigorous quality control is needed before they can be used in the clinic setting [41, 48]. Until now, the effects of cMSCs and subpopulations of EVs on AS have not been investigated.

Here, we intend to assess whether the subpopulations of EVs have a similar ability to recover reproductive function of a rat model of AS compared to cMSCs and heterogenous parental MSCs (hMSCs).

## Materials and methods

### Ethical statement

The Institutional Review Board and Institutional Ethical Committee of Royan Institute, Tehran, Iran (ethical code: IR.ACECR.ROYAN.REC.1398.073) approved the animal studies and procedures. In addition, all the animal experiments adhere to the ARRIVE guidelines.

### Animals

We obtained 76 nulliparous female Wistar albino rats (eight weeks old) that weighed 200–250 g from Royan Institute, Tehran, Iran. The rats were housed in the animal laboratory under standard conditions of 23 ± 2 °C, a 12-h light–dark cycle, and free access to water and food. After a one-week adjustment period, we began the interventions when the animals were in diestrus, which was confirmed by vaginal smears. The smears were obtained by daily collection of vaginal discharges each morning from the rats, for two consecutive weeks. The secretions were smeared on a slide and examined under an optic microscope. The phase of the estrus cycle was determined by specifying the ratio of epithelial cells, cornified cells, and leukocytes. Inclusion criteria included general health and having a regular estrous cycle confirmed by vaginal smear. If any injury, infection or secretion were observed on the skin surface or vaginal area, the animal was excluded. Also any abscess and inflammation (mild or severe) in the pelvic area and organs resulted in exclusion of the animal. The experimental unit was a single rat.

### Induction of the AS rat model

First, we induced the AS rat model by mechanical injury [49] to emulate uterine curettage in human [50]. This model was verified in two female Wistar albino rats as a pilot. We did not have human endpoints. All surgical procedures were performed in the diestrus phase of estrous cycle by the same researcher to eliminate bias from different individual’s techniques. All of these surgical steps were carried out under sterile condition in the animal laboratory. Briefly, adult female Wistar rats (200–250 g) were anesthetized with intraperitoneal (IP) injections of 3% medetomidine hydrochloride (Dorbene Vet; 1 mL/kg) and 75 mg/kg ketamine (Alfasan Diergeneesmiddelen BV). The animals were monitored until a deep anesthesia was reached. This was continued during surgery. Then rats were placed in the supine position and their abdomens were shaved, and a diluted iodophor solution was applied to the shaved area. Each laparotomy was performed under sterile surgical conditions. After a low abdominal midline incision, the uterus of each rat was exposed, and an ophthalmic scissor was used to excise the uterine wall (approximately one-third in length) vertically from each uterine horn. The inner surface of uterus was scraped with no. 22 surgical scalpel blades (Aesculap AG, Am Aesculap-Platz, 78,532 Tuttlingen, Germany) until the endometrial surface felt coarse, leaving the mesometrium intact. The uterine surface was then washed with a sterile saline solution. The uterine wall was closed with 8.0 polypropylene nonabsorbable surgical sutures (Deklene^®^ II, Teleflex Medical OEM). Following infliction of the mechanical damage, the uterus was returned to the abdomen, and the muscles and skin of the abdomen were sutured layer-by-layer with 5.0 vicryl absorbable suture (Ethicon, VCP392Z). After surgery and transplantation, each animal received an IP injection of 100 µl atipamezole hydrochloride (Alzane; 5 mg/ml) to reverse the sedative and analgesic effects of medetomidine.

Subsequently, the rats were placed on a warm stage at 37 °C to ensure recovery within 2 h. The recovered animals were returned to the animal laboratory. All rats received daily IP injections of a prophylactic antibiotic (enrofloxacin, 15 mg/kg, Rooyan Darou, Tehran, Iran) and subcutaneous (sc) injections of an analgesic (tramadol, 5 mg/kg, Alborz Darou, Tehran, Iran) for pain relief for three days after surgery. Animals were followed up for any sign of pain such as movement inability until scarifying, but no adverse effect was observed. Validity of the AS model was evaluated by comparison of histological staining of normal uterus sections with damaged uterus sections at two days and two weeks after generation of the AS model.

### Culture of cMSCs

We used isolated human bone marrow-derived cMSCs, which were obtained from the Royan Stem Cell Bank (RSCB0178), Tehran, Iran. These cells were prepared and characterized as we previously described in details [15]. Briefly, mononuclear cells were initially cultured at a low density to obtain cMSCs as the initiating cell material and improve the homogeneity of the final stem cell products. cMSCs were produced from single CFU derived colonies. Using cloning cylinders, the separate colonies were dissociated enzymatically using TrypLE (Thermo Fisher, USA). The cell suspension was then transferred to new tissue culture dishes. The selected colony was used at passage 8–11 in this study. The cells were cultured in low glucose DMEM medium (10,567,022, Thermo Fisher) supplemented with 10% fetal bovine serum (FBS, HyClone, USA) and 2 mM GlutaMAX (Life Technologies) at 37 °C and 5% CO_2_. The medium was renewed every 72 h. The confluent cells were passaged every five to six days.

### Isolation and characterization of human clonal mesenchymal stem cells-extracellular vesicles (cMSC-EVs)

cMSC-EVs were collected from conditioned medium. Our experiment was performed using 72 h conditioned medium of the cells (4 ml/1 × 10^6^ cells). The collected conditioned medium was centrifuged at 3000 g for 10 min to remove cell debris. The medium was centrifuged at 20,000 g for 30 min at 4 °C; then the pellet was suspended in PBS without Ca^2+^ and Mg^2+^ (PBS^−^, Thermo Fisher Scientific, 70013-032) and centrifuged at 20,000 g at 4 °C for 30 min (EV20K). To obtain a pellet from the second EV subpopulation, the supernatant was ultra-centrifuged at 110,000 g for 120 min at 4 °C. In the next step, the pellets were suspended in PBS^−^ and centrifuged by ultra-centrifugation at 110,000 g for 120 min at 4 °C (EV110K). The final pellets ((EV20K, EV110K) were resuspended in PBS^−^ and, after being snap frozen using liquid nitrogen, they were stored at − 80 °C for downstream experiments. According to MISEV guideline it would be better to measure two parameters for reporting quantity of EVs in a sample based on protein, particle, lipid or cell equivalence. Therefore, we normalized the amount of EVs based on protein dosage and number of the cells. We have isolated approximately 0.64 µg of EVs from every million cells. The morphology of the EVs was checked by scanning electron microscopy (SEM). For Western blot, the mouse anti-CD63 (Abcam: Ab8219; 1:500), mouse anti-TSG101 (Genetex; Gtx70255; 1:500) and mouse anti-CD81 (Santa cruz: SC7637; 1:500) surface markers as positive markers and Calnexin (1:500, Santacruz: Sc11397) as a negative marker were incubated overnight with the membrane. Protein content of the EVs was evaluated with a BCA Protein Assay Kit (Pierce, Thermo Fisher) and checked out by running on SDS-PAGE followed by staining by Coomassie Brilliant Blue (CBB). 20 µl EVs were loaded on SDS–page 10%, then transferred to PVDF membrane for western blotting. BSA (5%) was then used for blocking the blot for one hour at RT. The first antibodies were introduced in the next stage, which was done overnight at 4 °C. Then, the membrane was washed by TBST Special (1X) and incubated with an appropriate secondary antibody (Goat anti-Mouse IgG (31,437, Invitrogen, 1:50,000) and Donkey anti-rabbit IgG (K0810, Santa cruse, 1:50,000) for 1 h at room temperature. The membrane was washed, HRP-conjugated secondary antibodies was applied, and the bands were seen using the Gel Doc apparatus. The size distribution of the EVs was measured by dynamic light scattering (DLS, Malvern Instruments, Malvern, UK).

### Experimental design and treatment protocol

The animals (totally 76) were numbered and divided into the following seven groups based on table of random numbers. The surgeon was aware during allocation of the animals to intervention groups, collection and assembly of data. Other experimenter were blind to prevent subjective bias. The treatments were administered after inducing mechanical injury: (i) intact control (*n* = 8) received no intervention or treatment; (ii) sham surgery (*n* = 8) underwent abdominal surgery, and incisions and suturing were carried out on both horns; however, the curettage procedure was not performed; (iii) vehicle (*n* = 12) received 100 µl of medium in IV; (iv) hMSC (*n* = 12) received tail vein injections of 5 × 10^6^ hMSCs in 100 µl PBS; (v) cMSC (*n* = 12) received tail vein injections of 5 × 10^6^ cMSCs in 100 µl PBS; (vi) EV20K (*n* = 12) received intrauterine injections of 20 µg EV20K in 200 µl PBS per horn; and (vii) EV110K (*n* = 12) received intrauterine injections of 20 µg EV110K in 200 µl PBS per horn. We chose these concentrations of MSCs and EVs based on the previous similar reports [6, 20, 51].

### Labeling and ex vivo tracing of EVs

The EVs were labeled with a luminal fluorescent dye (Calcein AM, Invitrogen, c3099) to enable detection after they were injected into the animals. First, 50 µl EVs (1 µg/µl) were resuspended in 40 µl of the calcein AM (1 µg/µl) solution and the suspension was incubated at 37 °C for 30 min. The unincorporated dye was removed by using exosome spin columns (MW 3000). Immediately after removal of the excess dye, the mixture of EVs and calcein AM was infused into the uterine horns of the AS rat model. On days 0, 7, and 14 after surgery and the EV injection, the rats were sacrificed, and their uteri were collected. The presence of calcein AM labeled EVs in the uterine horns was visualized by UVI gel documentation (Uvitec, Cambridge, UK) and analyzed by UVI photo version Q9 alliance software (Uvitec, Cambridge, UK).

### Sampling

A total of four animals from each group were sacrificed at the end of the second week after the AS model induction and MSC transplantation. All animals were euthanized using CO_2_ for at least 5 min in euthanasia chamber. The animals were confirmed for lack of respiration and faded eye color. Then, the right uterine horns were harvested, snap frozen, and maintained at − 80 °C until needed for ELISA for protein expression analyses. The left horns were fixed in 10% formalin for histopathological examinations.

### Histopathological examinations

The tissues (*n* = 4/per group) were fixed in formalin, embedded in paraffin blocks after 48 h, and then sectioned into 6-µm transverse sections. After deparaffinization and rehydration of the uterine transverse tissue sections, hematoxylin and eosin (H&E) and Masson’s trichrome (MT) stainings were performed.

For Masson staining, five micrometer tissue sections were deparaffinized and rehydrated through 100% alcohol, 95% alcohol, 70% alcohol, so were washed in distilled water. The sections re-fixed in Bouin's solution for 1 h to augment staining quality. To remove yellow color, they were taken under running tap water for 5–10 min. After staining in Weigert's iron hematoxylin working solution for 10 min, sections were rinsed in running warm tap water for 10 min and were washed in distilled water. Subsequently, sections were placed in Biebrich scarlet-acid fuchsin solution (Biebrich scarlet: 90 ml 1% aqueous, acid fuchsin: 10 ml 1% aqueous solution, acetic acid: 1 ml glacial) for 10–15 min. Washing in distilled water was repeated, and then they were placed in phosphomolybdic–phosphotungstic acid solution (Phosphomolybdic acid: 25 ml 5% aqueous solution, Phosphotungstic acid: 25 ml 5% aqueous solution) for 10–15 min. Then, the sections were transferred directly (without rinse) into aniline blue solution for 5–10 min and rinsed briefly in distilled water and differentiated in 1% acetic acid solution for 2–5 min and were washed in distilled water. Since very quickly dehydration through 95% ethyl alcohol, sections were cleared in xylene and were mounted with resinous mounting medium.

Structural changes in the uterine tissues were observed and some of the morphometric parameters were calculated using an Olympus BX51 microscope (Japan). Microphotographs were captured using an Olympus DP70 camera (Japan). The instrument was equipped with Olympus UPlanFLN (Japan) objective lenses, Olympus U-MWU2 and U-MWG3 filters and OLYSIA BioReport as an acquisition software. The images were acquired in 200 dpi (for H&E/ MT images) and 150 dpi (for immunofluorescent images) resolution. We increased exported resolution to 300 dpi in Adobe Photoshop CC 2017.

Sections from the beginning, middle, and end of the uterine horns from each animal were examined. The number of endometrial glands in each 3600 µm^2^ area of 3 transverse sections belong to left uterine horn from each animal were counted. Endometrial thickness was measured by ImageJ software (version 1.46r, http://imagej.nih.gov/ij/). The thickness was estimated based on the ratio of the endometrial diameter to the total section diameter. The average endometrial thickness was calculated from two sections per slide and from five slides per animal. Then, the percentage of the fibrotic areas was determined by ImageJ software. We applied a grading system based on color intensity to do an accurate calculation; light blue (minor grade) which indicates a lower number of collagen fibers, moderate blue as a mild fibrosis, and dark blue (major grade) which indicates severe accumulation of collagen fibers. The average area of collagen fiber deposits was calculated with the 2 sections.

### Immunofluorescence and immunohistochemistry assay

For immunofluorescence staining, the uterine sections (*n* = 4/per group) were deparaffinized, then rehydrated in decreasing concentrations of ethanol (100% to 70%) and water. The slides were subsequently transferred to a sodium citrate buffer (Sigma-Aldrich s1804; pH 6) and placed inside an antigen retrieval device. The sections were washed three times with 0.05% PBS-Tween for 5 min. In the case of immunohistochemistry (IHC), endogenous peroxidase was blocked by immersion in 0.3% hydrogen peroxide for 20 min at the room temperature and then section was rinsed with PBS-Tween again. Subsequently sections were permeabilized with 0.5% Triton X-100 at room temperature for 40 min, blocked with 5% bovine serum albumin (BSA) for 1 h at 37 °C. Next, were incubated overnight with primary antibodies alpha smooth muscle Actin (1:500, Abcam, ab7817), a marker of fibrosis, STEM 121 (1:200, Dontech, Y40410), a human marker to detect human cMSCs [52], and Ki67 (1:500, Abcam, ab15580) for detection of endometrial cell proliferation. Afterward, the sections were incubated with secondary antibodies (1:500, ab150117, 1:500, Invitrogen, Abcam, ab98509, DyLight^®^ 594) for 1 h at 37 °C, counterstained with DAPI. DAB was used as chromogen in IHC sections and then counterstained with hematoxylin. Negative and IgG controls were performed using PBS and Isotype control antibody (STEM 121: Ab178000, Ki67: Ab37415) instead of primary antibody, respectively. The sections were observed under a fluorescence microscope (Olympus BX51). At least four fields and an average of 2000 cells were counted at 20 × magnification to assess the amount of cell proliferation in the sections with Photoshop software.

### ELISA assay

Local concentrations of vascular endothelial growth factor (VEGF), leukemia inhibitory factor (LIF), tumor necrosis factor-*α* (TNF-*α*), and interleukin-10 (IL-10) were measured in the uterine tissues (*n* = 4/per group). For this purpose, tissue samples were collected and evaluated by commercial ELISA kits according to the manufacturers’ protocols to determine the amounts of VEGF (R&D, RRV00), LIF (LSBio, LS-F13295), TNF-*α* (R&D, RTA00), and IL-10 (R&D, R1000) in the tissues.

For protein extraction from rat uteri, we initially dissected the tissues and washed them using ice cold PBS, to remove the blood. Then, tissue was catted into smaller pieces and kept on the ice. We harvested the same amount of each tissue (10 mg) to electric homogenizer and added additional 500 µL of RIPA buffer (Cat No: 9806S, cell signaling) with protease inhibitors Cocktail (Cat No: 5871 s, cell signaling) during homogenization. Then, completely homogenized contents, then centrifuge it in microcentrifuge tubes at 14,000 × g for 20 min. Finally, we collected the supernatant in fresh tube and used them for next steps.

To perform sandwich ELISA, all reagents, standard solution, and samples were prepared according to the kit instructions and kept them at room temperature for 20 min. Then, added 50 µL of diluent solution to each microplate. Afterward added 50 µL (for TNF-*α*, IL-10, and VEGF) and 100 µL (for LIF) of sample and standard solution and incubated them for 2 h. The plate then was emptied and 100 µL of Detection Reagent A solution was added. Incubation at 37 °C for 1 h was performed (This stage is just for LIF). Again, we emptied the plate and washed them 3 times each time with 400 µL of washing solution. 100 µL of the desired conjugate solution (TNF-*α*, IL6 and IL10) was added to each well. Incubation at room temperature for 2 h was added. But for LIF in this stage, we added 100 µL of Detection Reagent B solution. Then incubated it at 37 °C for 1 h. We emptied the plate and washed them 3 times each time with 400 µL of washing solution. Then added 100 µL of the substrate solution (but 90 µL for LIF) to each well and incubated them in the dark for 20 min. Afterward 100 µL of Stop solution was added to each well. Finally, using the ELISA reader, we read the amount of light absorption at 450 nm and imported the data into excel and drew the standard curve based on OD value of the sample.

### Functional reproduction test

We used the mating test to evaluate the ability of each endometrium for pregnancy and ability to produce healthy offspring. Female rats from all the experimental groups (*n* = 4–8) were allowed to mate with fertile males (2:1 female to male ratio) and the animals were followed for ten weeks. The number of deliveries and pups were assessed.

### Statistical analysis

PASS software was used to outline the sample sizes. No animals or data points were excluded from the analysis. The GraphPad PRISM 8 software was applied to analyze data. The results were reported as mean ± standard deviation (SD). Means of samples from the experimental groups were compared by one-way analysis of variance (ANOVA) and post hoc Tukey test. *P* < 0.05 indicated statistical significance. Non-normally distributed data were evaluated with the Kruskal–Walli’s test.

## Results

### Characterization of cMSC-EVs

**Fig. 1:**
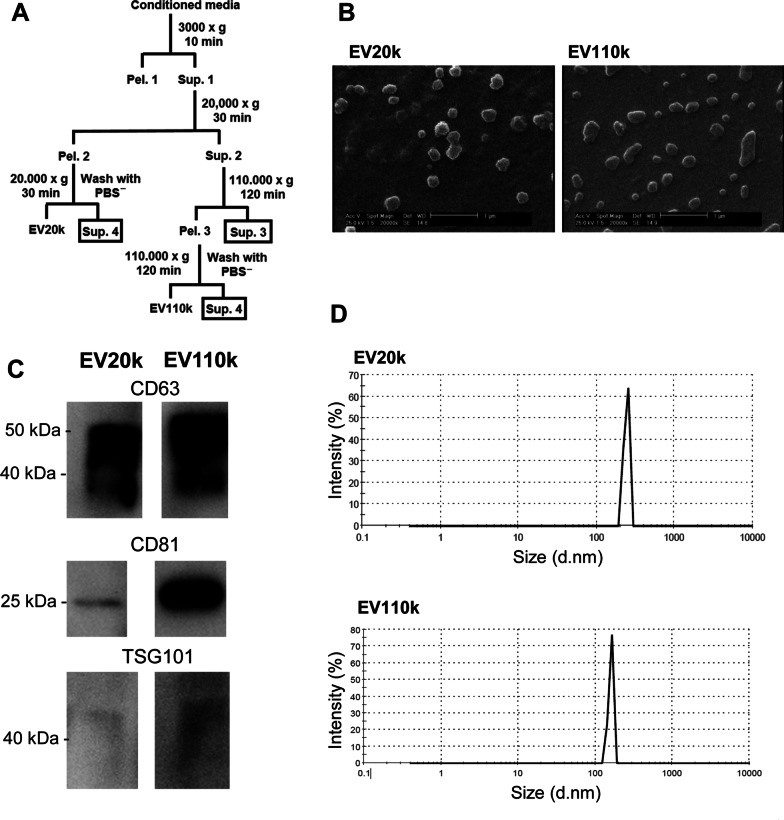
Isolation and characterization of clonal mesenchymal stromal cell (cMSC)-derived extracellular vesicles (EVs). **A** Centrifugation steps for isolation of EVs. **B** Scanning electron microscopy (SEM) of the EVs morphology. **C** Western blot analysis of the EVs enriched proteins (CD63, CD81, and TSG101). Full-length blots are presented in Additional file 1: Fig. S1. **D** Analysis of EVs size distribution by dynamic light scattering (DLS)

The human cMSCs and hMSCs were homogenous populations that spindle-shaped morphologies. To prepare the EVs, we used MSC conditioned media (Fig. 1A). The EVs that we prepared by centrifugation at 20,000 g or 110,000 g during steps three and four were named EV20K and EV110K, respectively (Fig. 1A). The final sediments that contained EVs were resolved in up to 100 µl of sterilized PBS and kept at − 80 °C.

We used PBS buffer for cryopreservation of both subpopulations of EVs. These EVs were characterized after thawing. SEM assessment showed that these isolated EVs had a spheroidal morphology (Fig. 1B). The protein extracted from EVs was loaded on SDS-PAGE and stained with Coomassie Brilliant Blue (CBB) and confirmed the protein content estimation by BCA (Additional file 1: Fig. S1A). Western blot analysis indicated that both EVs expressed the enriched proteins TSG101, CD63, and CD81 (Fig. 1C and Additional file 1: Fig. S1B); however, no calnexin was detected (Additional file 1: Fig. S1C). DLS assessment indicated that the EVs were 120–400 nm in diameter and EV20K was slightly larger than EV110K (Fig. 1D).

### Transplantation of MSC or MSC-EVs improved endometrial structure and function in rat model for AS

To test the in vivo functionality of the off-the-shelf prepared cMSC-EVs, we injected them intraluminally into both horns. We chose local administration of these EVs to increase their efficiency because of the rapid elimination of systemic injections of EVs from peripheral circulation [53]. The MSCs were transplanted through the tail vein.

**Fig. 2:**
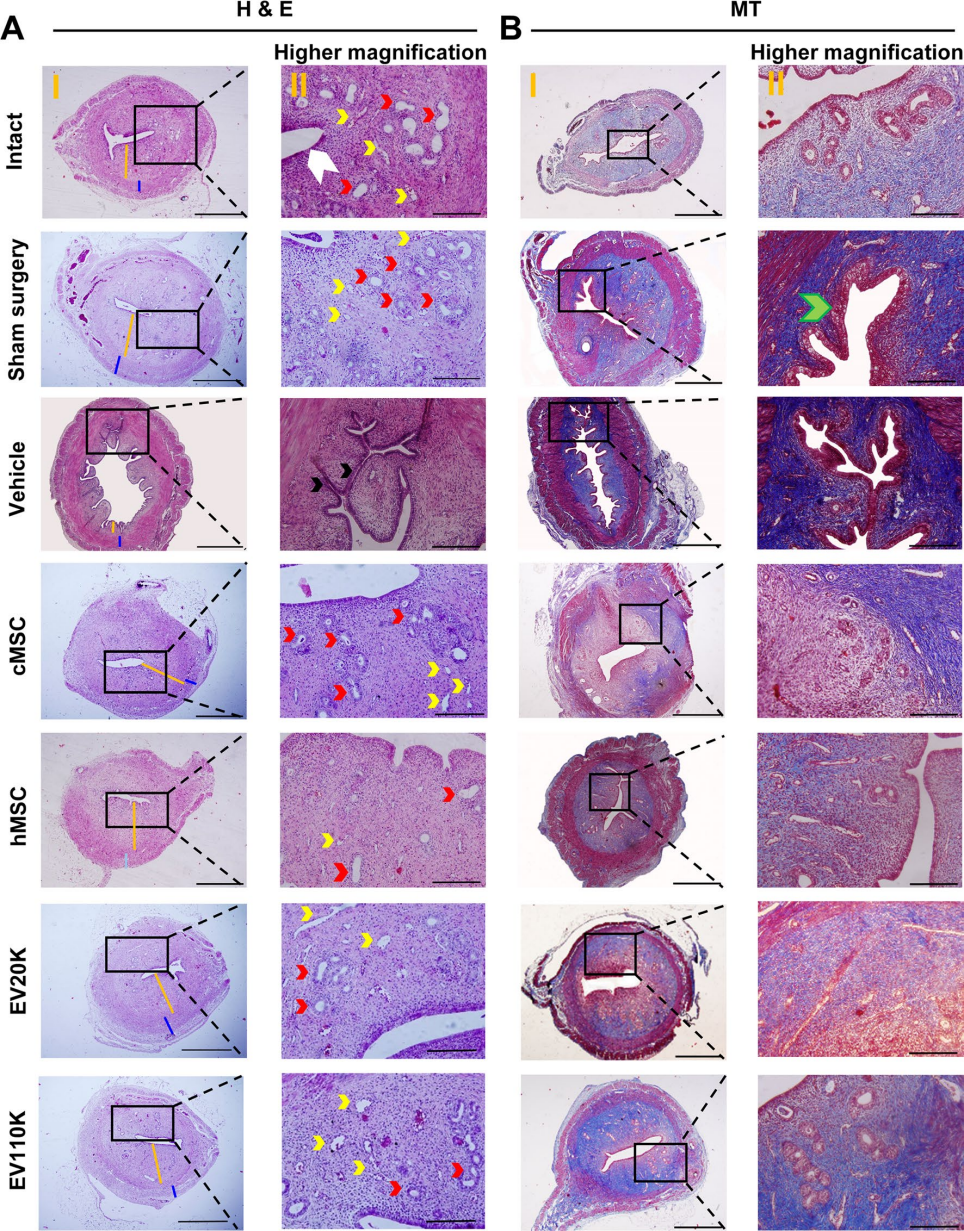
Histology of the uterine transverse sections in the treatment groups. **A** Hematoxylin and eosin (H&E) staining. The endometrial surface is covered with simple high columnar epithelial cells (white arrowhead); abundant blood vessels (yellow arrowheads); numerous endometrial glands (red arrowheads); and normal uterine cavity are noted in the intact group versus the vehicle group with uterine wall adhesion (black arrowheads), avascularization of stromal tissue, decreased ratio of the endometrium to the myometrium (orange line: endometrium; blue line: myometrium), and abnormal epithelium (green arrowhead). These morphological features were improved in the clonal mesenchymal stem cell (cMSC), heterogenous parental MSC (hMSC), EV20K, and EV110K groups. **B** MT staining showed mechanical damage increased level of collagen fiber depositions (more areas with dark blue: indicator of collagen fibers intense accumulation) severely in vehicle group comparing intact, but cMSC, hMSC, EV20K, EV110K administration diminished fibrotic area in the endometrium (more areas with light blue: indicator of minor mass of collagen fibers). I: Scale bar 1000 µm, II: Scale bar 200 µm

**Fig. 3:**
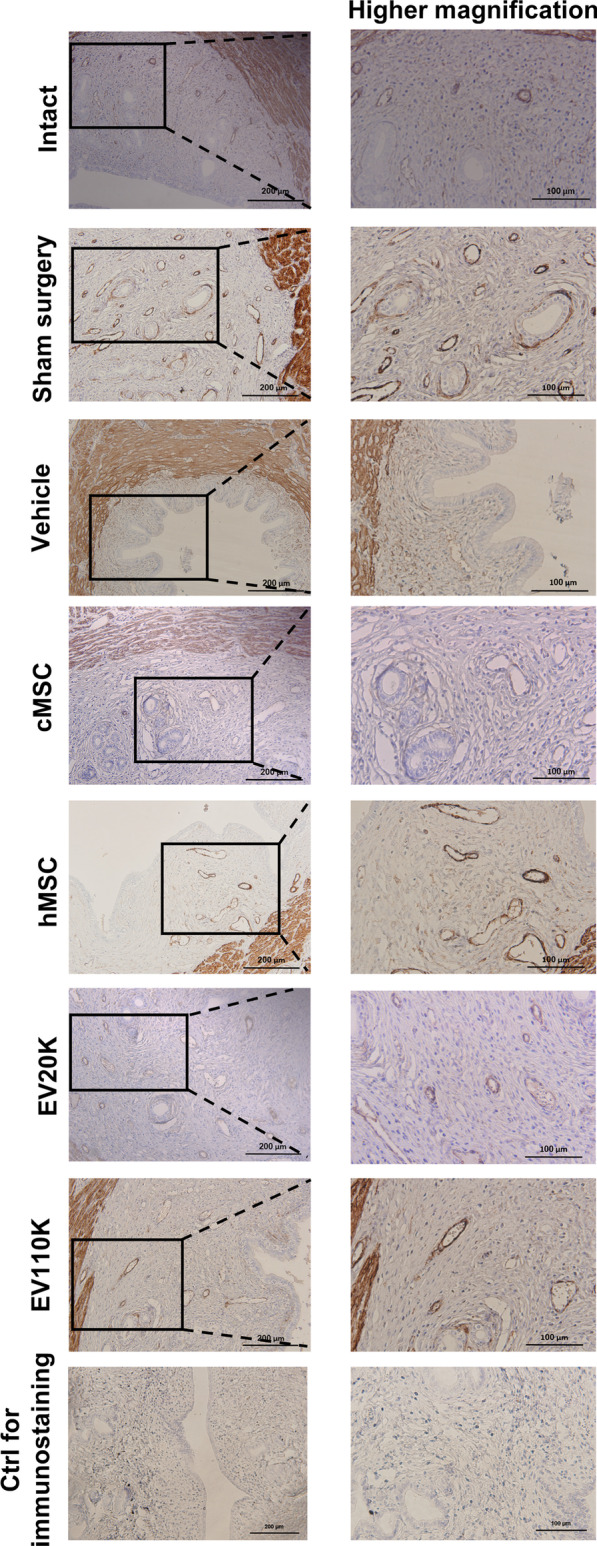
Endometrial fibrosis after MSCs and EVs treatment. Immunohistochemistry staining shows the decrease of intercellular and periglandular expression of *α*-SMA in the MSCs- and EVs- treated uterine compared to vehicle and sham surgery groups

At 14 days after infliction of the mechanical endometrial injury, we observed the formation of a few fibrous adhesions (Additional file 2: Fig. S2) as demonstrated by the increased extracellular matrix (ECM) collagen depositions and scattered and decreased endometrial glands in the uterine cavities, which were confirmed by H&E (Fig. 2A, Additional file 3: Fig. S3), MT staining (Fig. 2B, Additional file 4: Fig. S4), and *α*-SMA expression (Fig. 3).

**Fig. 4:**
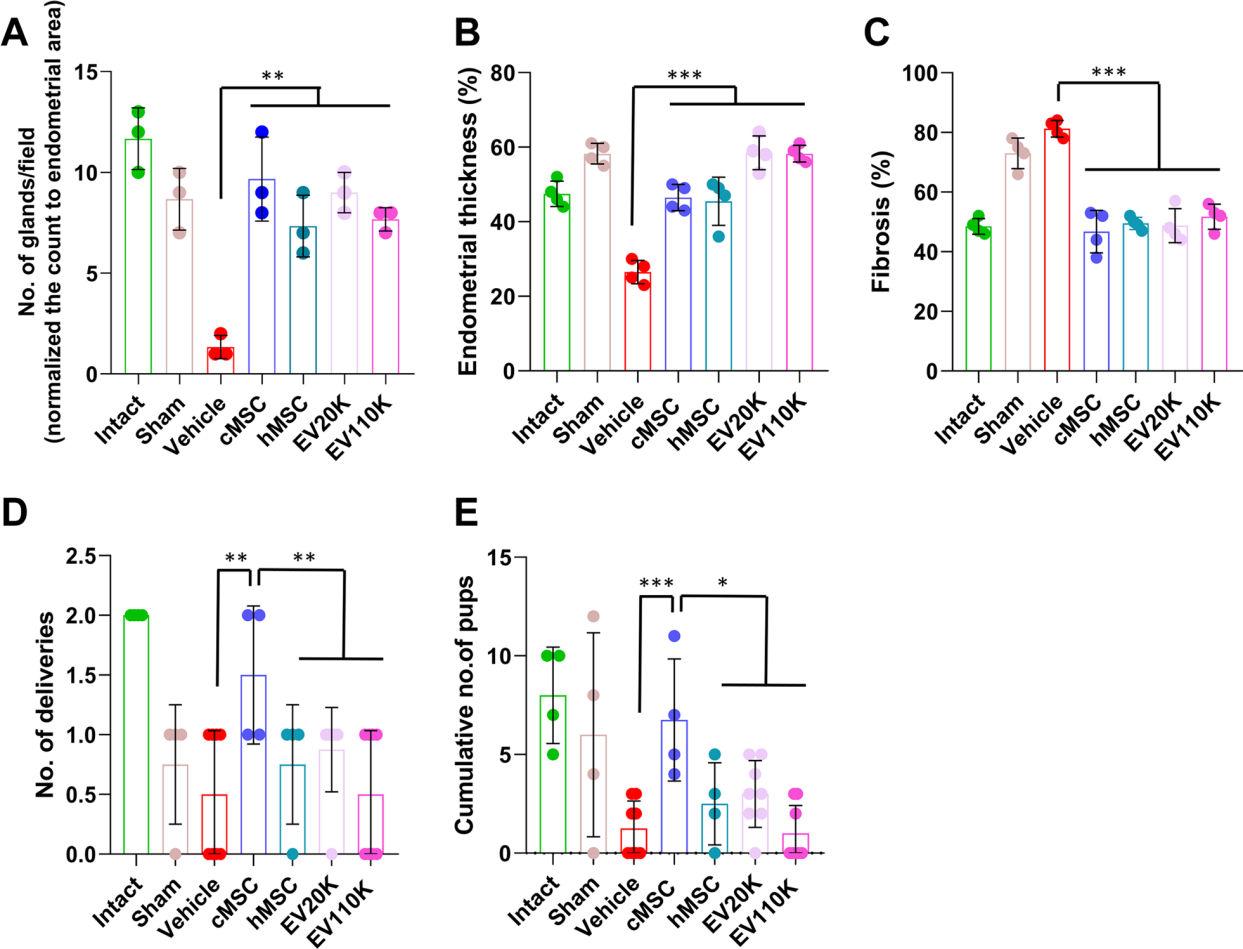
Quantification of histological staining and fertility. The comparison analyses of **A** endometrial gland per field, **B** endometrial thickness, **C** fibrosis percent, **D** the number of deliveries, and **E** the cumulative number of pups between all groups

Histological analysis two weeks after transplantation showed that the number of glands per area and endometrial thicknesses in the vehicle-treated animals (1.3 ± 0.5 and 26.5 ± 3.1%) were lower compared to the intact (11.6 ± 1.5 and 47.5 ± 3.4%) and sham surgery animals (8.6 ± 1.5 and 58.2 ± 2.7%) (*P* < 0.001). However, they were improved in the groups transplanted with cMSCs (9.6 ± 2.0 and 46.5 ± 3.5%) and hMSCs (7.3 ± 1.5 and 45.5 ± 6.4%), and in the groups that received both EV subpopulations (EV20K (9.0 ± 1.0 and 58.5 ± 4.5%) and EV110K (7.6 ± 0.5 and 58.2 ± 2.2%) (*P* < 0.05) (Fig. 4A and B). Moreover, the fibrotic area ratio increased in the vehicle-operated uteri (81.2 ± 2.7%) in comparison with intact uteri (48.5 ± 2.6%) (*P* < 0.001) and decreased after injections of cMSCs (46.7 ± 7.0%), hMSCs (49.5 ± 2.0%) or both EVs (EV20K: 48.7 ± 5.7% and EV110K: 51.7 ± 4.1%) (*P* < 0.001) (Fig. 4C). *α*-SMA expression also confirmed fibrosis. While, *α*-SMA highly expressed in the stroma, perivascular, and periglandular areas of the endometria of vehicle and sham surgery groups, it was decreased MSC- and EV-treated animals (Fig. 3).

We also assessed functional recovery in the AS animals following transplantation of MSCs or injection of EVs by observing the delivery (Fig. 4D) and cumulative number of pups (Fig. 4E) per animal. The animals were allowed to mate (2:1 female to male ratio) two weeks after transplantation of the cMSCs or injection of the EVs. In the intact and sham surgery groups, 100% and 75% of animals conceived. However, the number of deliveries improved after the cell transplantations of cMSCs (100%, *P* < 0.05) and hMSCs (75%), or administration of EV20K (87.5%) and EV110K (75%) compared to the vehicle-treated animals (50%) (Fig. 4D).

Furthermore, the cumulative number of pups per animal was calculated ten weeks after transplantation. The cumulative number of pups in the cMSC group (6.7 ± 3.0%) improved in comparison with the other groups (*P* < 0.05, Fig. 4E).

**Fig. 5:**
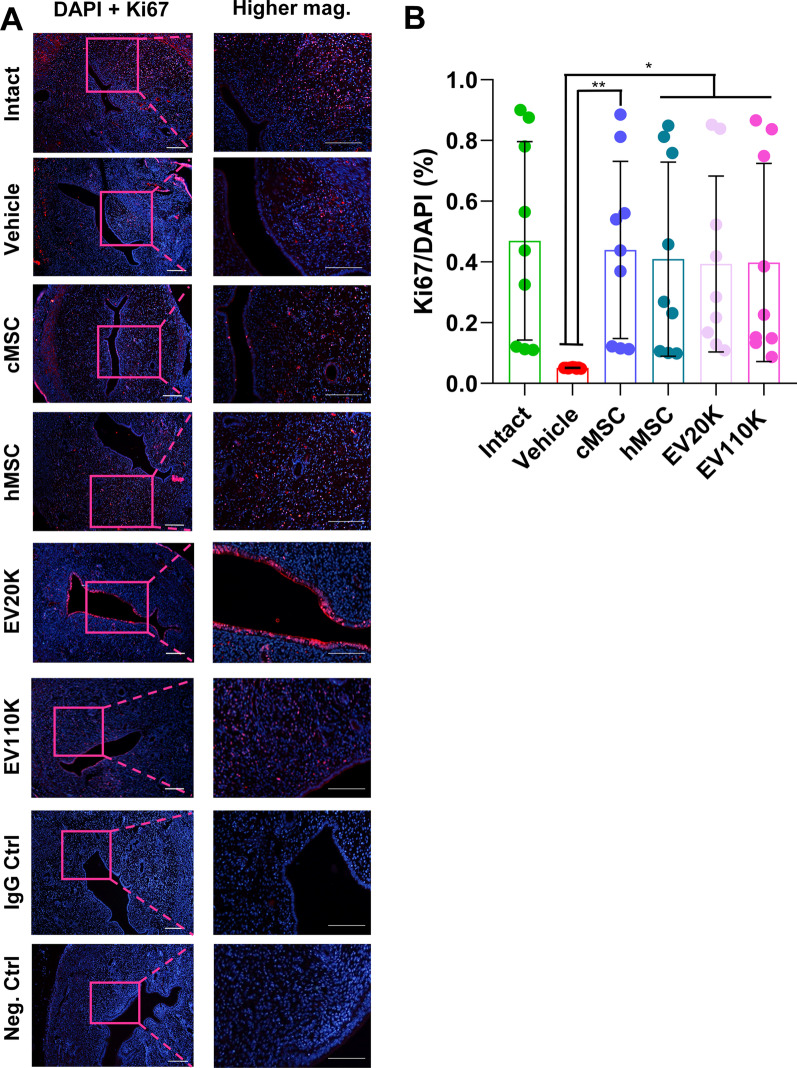
Cell proliferation in the endometrium. **A** Immunofluorescence staining for proliferation marker Ki67. **B** Statistical analysis showed more Ki67 positive cells in the clonal mesenchymal stem cell (cMSC), heterogenous parental MSC (hMSC), EV20K, and EV110K groups compared with the vehicle group. Scale bars: 200 µm

Immunofluorescence analysis for the Ki67 proliferation marker indicated a decrease in the number of positive cells in vehicle-operated uteri (0.05 ± 0.00) in comparison with intact animals (0.47 ± 0.33%) (*P* < 0.03); however, it was preserved after the cell transplantations or EV injections (EV20K: 0.40 ± 0.29%, EV110K: 0.40 ± 0.32%) (*P* < 0.05, Fig. 5). Interestingly, in the EV20K group, this increase was mainly seen in the epithelial compartment. In the other groups, proliferation was mostly observed in the stromal sections.

Together, a lower gland number, thinner endometria, increased fibrotic areas, and lower proliferation of epithelial and stromal of uteri were observed in vehicle-treated uteri compared with intact and sham-operated animals, which suggested that the endometrial morphologies failed to fully regenerate two weeks after the injury, whereas the evaluated parameters improved after transplantation of both cell types (cMSC and hMSC) or both EVs subpopulations (EV20K and EV110K). However, cMSCs were more efficient than both fractions of EVs and hMSCs in restoration of reproductive function.

The feasibility of EV20K production that facilitates the use of EV20K for translational purposes and permits extensive and repeated product generation and testing prior to clinical use encouraged us to continue the remainder of the experiments with EV20K and compare them with cMSCs.

### Location of transplanted MSCs and EVs

**Fig. 6:**
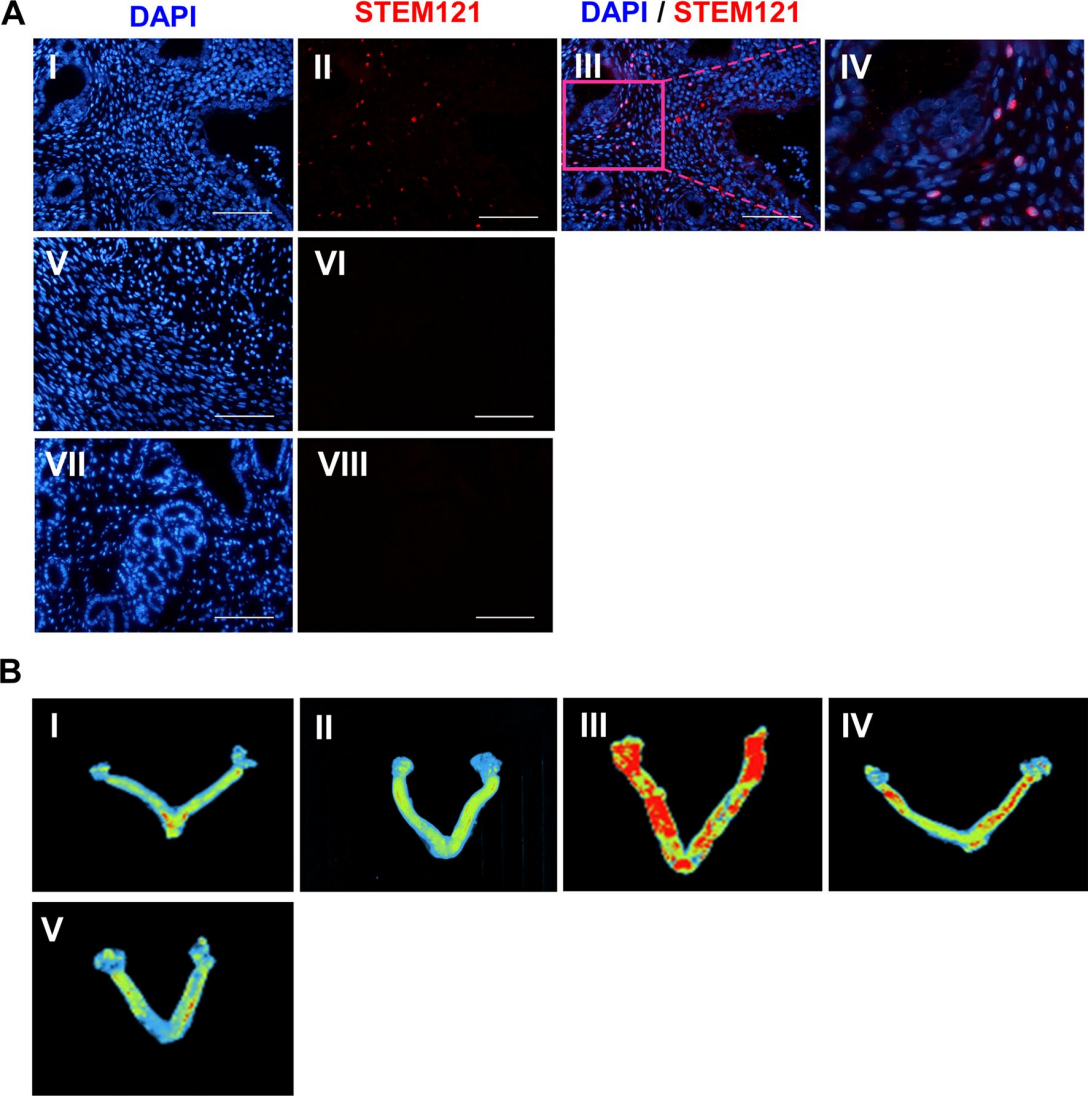
Tracking of injected clonal mesenchymal stem cells (cMSCs) and extracellular vesicles (EVs) in the treated uterine horns. **A** STEM121 immunofluorescence staining was used to detect transplanted clonal mesenchymal stem cells (cMSCs) in the endometrial tissue (I–IV); IgG control (V–VI), negative control (VII–VIII). Scale bar: 100 µm. **B** Labeling and ex vivo tracking of extracellular vesicles (EVs) by Calcein AM dye after in situ administration in the uterus at different time points: (I) Intrauterine injection of unlabeled EVs as a negative control. (II) Intrauterine injection of Calcein without EVs as staining control. Intrauterine injection of Calcein-labelled EVs at Day 0 (III), 7 day (IV), and 14 days (V) after EV treatment. The presence of Calcein in the uterine horns was visualized with UVI gel documentation

We used immunostaining for the human specific antibody for STEM-121 to trace the transplanted MSCs in the uteri. The results indicated that the cMSCs migrated into the stromal uterine tissue (Fig. 5). Considering the challenges of lipophilic dye (such as PKH and DIR) labeling of EVs [54, 55], we preferred to label these EVs with calcein AM. Calcein AM is a non-fluorescent stain that activates after it enters the EVs. The activated calcein AM subsequently becomes fluorescent [56]. Tracing of calcein AM labeled EVs demonstrated that the EVs accumulated inside the uteri up to one week after the injection (Fig. 6).

### Effects of MSCs and EVs transplantation on cytokine expression

**Fig. 7:**
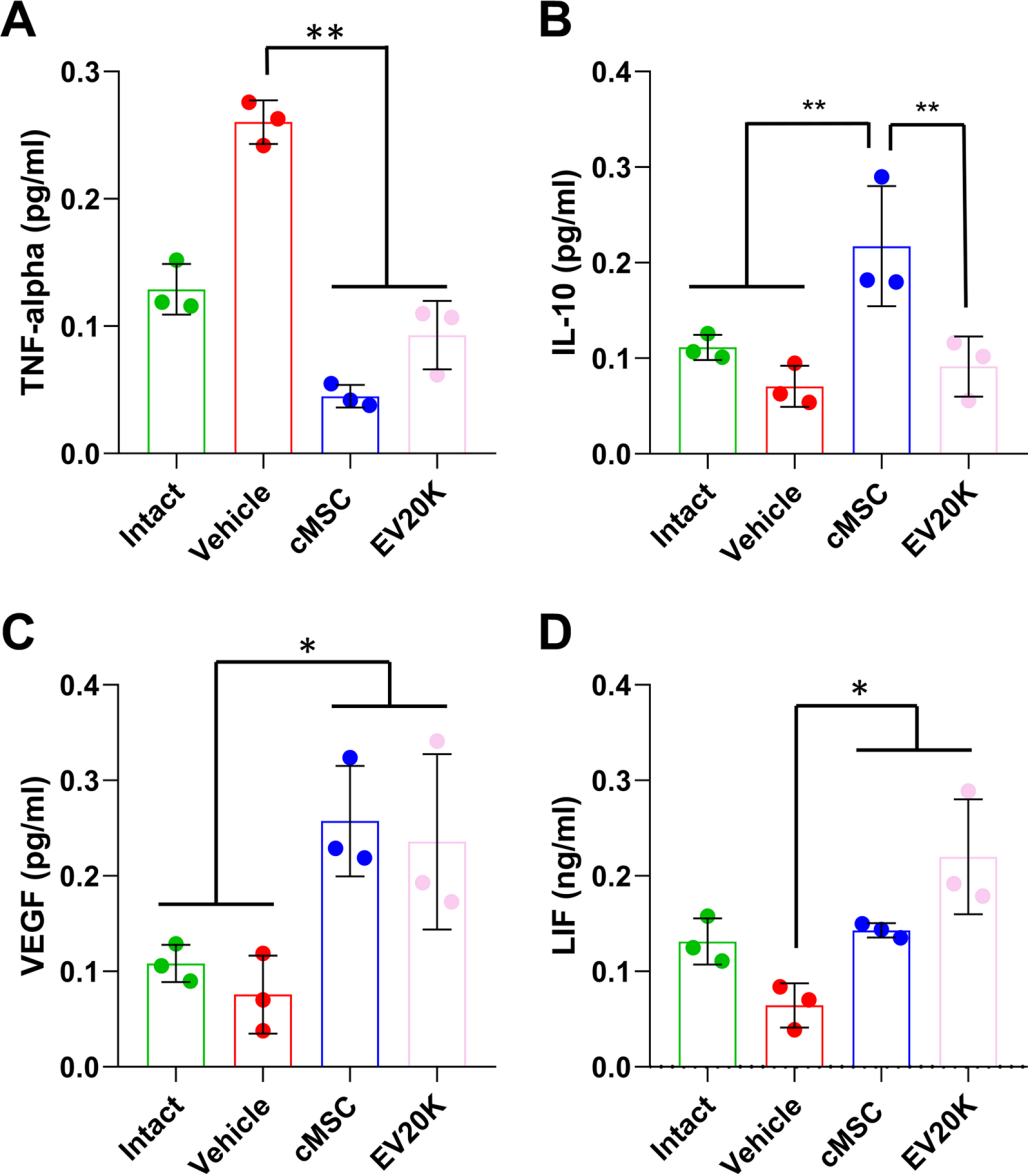
ELISA assessment of protein expression in the treated uterine tissue. Transplantation of clonal mesenchymal stem cells (cMSCs) and EV20K promoted endometrial regeneration via decreasing inflammation TNF-*α* as a pro-inflammatory cytokine (**A**) and IL-10 as an anti-inflammatory one (**B**), and by increasing angiogenesis (**C**) and receptivity (**D**)

MSCs migrate to an injured site and secrete proinflammatory factors, anti-inflammatory cytokines, and growth factors. ELISA was used to analyze expressions of the proinflammatory factor TNF-*α* [57] and anti-inflammatory cytokine IL-10 [58] in the uteri of the groups (Fig. 7). The expression of the proinflammatory factor TNF-*α* was lower in the cMSC (0.045 ± 0.009 pg/ml) (*P* < 0.05) and EV20K groups (0.093 ± 0.027 pg/ml) (non-significant) and IL-10 expression was enhanced in the uteri from the cMSC group (0.217 ± 0.063 pg/ml) compared to the vehicle group (0.071 ± 0.021 pg/ml). Assessment of VEGF expression indicated that the cMSC (0.257 ± 0.058) and EV20K treated uteri (0.236 ± 0.091 pg/ml) had significantly higher expression in comparison with the vehicle uteri (0.076 ± 0.040) (*P* < 0.05). The protein level of LIF, a marker for endometrial receptivity [59], was upregulated more in the cMSCs (0.143 ± 0.007) and EV20K animals (0.220 ± 0.060) compared to the vehicle animals (0.064 ± 0.023) (*P* < 0.05).

## Discussion

In this study, a rat endometrial mechanical injury model was used to assess the effects of immediate administration MSCs and EVs in prevention of AS. According to the results, both the cells and EVs could repair the injured rat endometria.

While, we treated our animals immediate after mechanical injury as other studies were reported [49, 60–63], there are some reports that transplanted cells or EVs, 1–30 days after modeling [21, 64–67].

Until now, MSCs from different sources such as bone marrow, adipose and placental tissues, and umbilical cord blood (for review see [68]) have been used in animal models of AS [21, 49, 60–67, 69–71]. Moreover, the therapeutic potential of MSCs has been documented in clinical trials of women who have unresponsive thin endometria caused by AS [72] or recurrent uterine adhesions [73]. However, these studies used classical hMSCs. The heterogeneity in classical hMSCs results in the variations of outcomes [11, 12]. Therefore, in this study we used cMSCs which are a homogenous population of MSCs that are generated from a single CFU by sub-culturing [15]. We have reported that these cMSCs express specific surface markers that have a differentiation potential [15] in accordance with the features of the International Society for Cell Therapy [34]. In this study, we demonstrated more deliveries and pups in the treated rats that received cMSCs in comparison with hMSCs. The positive effects of cMSCs have been reported in different animal models of inflammatory bowel disease, osteoarthritis, spinal cord injury, diabetes, atopic dermatitis, and pancreatitis [74–79].

By STEM121 labelling, we found that cMSCs are localized to the damaged endometria, which likely resulted in the regeneration of functional endometria. The exact mechanism that underlying MSCs function to improve endometrium remains unknown. To date, the therapeutic potential of transplanted MSCs to treat certain inflammatory disorders and destructive diseases could be related to mechanisms that involve secretion of bioactive molecules either directly or through EVs.

The regeneration process of an injured endometrium involves many events, including cell proliferation [66]. We observed increased cell proliferation in the endometria of AS rats that received the MSC transplantation. Cell proliferation is required for endometrial function [66] and the increase in cell proliferation following cell transplantation in our study indicated an improvement in endometrial function. Our finding is in line with previously reported proliferative effects of MSC in the endometria of an AS rodent model [57, 61, 80, 81].

In contrast to MSCs, MSC-EVs exhibit similar functions as the parent cells in addition to higher biological stability and lower immunogenicity [46, 82, 83]. Ultracentrifugation is the conventional method used to isolate EVs [34, 42]; however, the resultant EVs are a heterogenous population because of the overlaps in density or size of the different EV types [84]. Therefore, we used two centrifugation speeds (20,000 g and 110,000 g) to isolate two subpopulations of EVs (EV20K and EV110K), which were subsequently evaluated in an AS rat model. We cryopreserved the isolated EVs in PBS buffer. Both subpopulations of our EVs met the MISEV2018 guidelines [34] in terms of morphology, size distribution, and marker expression. The results showed that both were equally effective as seen by the improved gland numbers, endometrial thickness, and reduction in endometrial fibrosis. Moreover, the cell proliferation in the endometria of AS rats that received both types of EVs increased.

By calcein tracing, we found that the EVs localized to the damaged endometria, which likely led to the repair of functional endometria.

Since ultracentrifugation-based EV isolation has many drawbacks, which include the cumbersome nature of the ultracentrifugation process [85] and difficulties in GMP compatibility [85, 86], many reports have proposed the use of subpopulations of EVs that were isolated by centrifugation at speeds lower than 100,000 g [85, 87–89]. Based on our promising results, EV20K appears to be more feasible in terms of isolation, particularly in good manufacturing practice (GMP) facilities, because of a lower cost compared to EV110K. Therefore, we evaluated the most valuable factors that affect embryo implantation the endometria receptivity after administrating with cMSCs and EV20K in comparison with controls using ELISA.

Upregulation of VEGF during embryo implantation is useful to improve endometrial receptivity, early formation of blood vessels, and facilitate embryo adhesion [90, 91]. LIF is also involved in implantation processes such as uterine preparation for implantation, embryo-endometrial interaction, and trophoblast invasion [91, 92]. Our findings showed a significant increase in endometrial receptivity of cMSCs- and EV20K-treated animals, which was demonstrated by upregulation of VEGF and LIF. This agreed with the significantly increased numbers of deliveries and cumulative pups after the cMSC transplantation. These findings supported the results of previous studies that reported upregulation of VEGF and LIF in response to MSCs or EVs transplantations [19, 81]. Moreover, our experimental results showed that cMSC and/or EV20K administration led to significant downregulation of protein expression of TNF-*α*, a pro-inflammatory factor and upregulation of the anti-inflammatory factor IL-10. Therefore, these bioactive molecules which secret either directly by MSCs or transfer through EVs repair endometrial injury of an AS animal model through alleviating endometrial fibrosis and inflammation, enhancement of endometrial cell proliferation and angiogenesis, regulation of molecular markers related to endometrial receptivity, and improved fertility [19–21, 49, 67, 69–71].

Although we have observed the therapeutic potential of EV20K in the AS rat model, further studies are required to improve its efficacy. For example, the possibility of a second or third dose of MSCs or EVs might consolidate the initial effects. Further assessment of sustained release of the MSC-EVs through a hydrogel might determine if it could improve the therapeutic effects of EVs in an AS model. We observed that an IP injection of the mixture of a clickable polyethylene glycol (PEG) hydrogel with MSC-EVs resulted in extended accumulation of EVs in the liver of in a rat model of chronic liver fibrosis, and the histology of the livers revealed superior antifibrosis, anti-apoptosis, and regenerative effects of the EVs compared to the conventional bolus injection or free-EVs [53]. One of the limitations of our study is to assess whether rats with injured uterine have any changes in their sexual behavior that affects their coupling rate with males. Moreover, to produce a dose of EVs that are comparable to the action of the cells from which they are derived we need more MSCs or multiple EV harvesting (e.g., in a clinical trial for GVHD [44], a dose of EVs derived from four times of MSC population).

## Conclusions

These results show that cMSC and their derived “off-the-shelf” EV20K transplantations in a rat model of AS helped to repair the endometrial injury and restored reproductive more effectively than their hMSCs. We also confirmed that this off-the-shelf and GMP-compatible product (EV20K) might provide a feasible strategy to prevent AS. The strategy of using subpopulations of MSCs and EVs might open a new paradigm to extend the effects of disease targeting EVs.

## Supplementary Information

**Additional file 1: Fig. S1. **Full-length Western blots and SDS-PAGE of proteins extracted from EVs. (A) The protein extracted from EVs was loaded on SDS-PAGE and stained with Coomassie Brilliant Blue (CBB) and confirmed the protein content estimation by BCA. The resulting SDS-PAGE (red box) confirmed distinct protein profile of the EV20k and EV110k. The blue box represented the remain part of the gel that is related to other samples not related to this manuscript. (B) Full-length Western blots of corresponding bands of CD63, CD81, and TSG101. (C) The expression of Calnexin as a negative marker was tested and cMSCs lysate were used as positive control for Calnexin expression. The equal amount of proteins from two samples (EV20k and EV110k) were loaded into the SDS-PAGE and blotted based on detailed protocol in the method session and the blots were detected by antibodies. The resulting blot (red box) represent the whole-body image of blot and the dotted box represent the cropped band for the Figure 1C. The grey box related to other samples blot (not related to this study) that were run in the same gel.

**Additional file 2: Fig. S2**. Establishment and confirmation of the Asherman syndrome (AS) model in Wistar rats. (A) The surgical procedure. I. A vertical incision (approximately 1.5 cm) was made in the lower abdomen to expose the uterine horns. II. Excision of two-thirds from each uterine horn wall. III. Curettage was performed by scratching the inner uterine surfaces until the uterine walls became rough and pale. IV. Suturing and wound closure. (B) Histopathology of uteri in the normal and the AS model two weeks after surgery to ascertain the amount of endometrial damage. Hematoxylin and eosin (H&E) and Masson’s trichrome (MT) staining. Scale bar in the left side: 1000 µm, scale bar in the right side: 200 µm.

**Additional file 3: Fig. S3**. Hematoxylin and eosin (H&E). Scale bar in the left side: 1000 µm, scale bar in the right side: 100 µm.

**Additional file 4: Fig. S4**. Masson’s trichrome (MT) staining. Scale bar in the left side: 1000 µm, scale bar in the right side: 100 µm.

## Data Availability

All data generated or analyzed during this study are included in this published article.

## Abbreviations

AS: Asherman’s syndrome
CFUs: Colony forming units
cMSCs: Clonal MSCs
EV110K: 110,000 G-derived EVs
EV20K: 20,000 G-derived EVs
EVs: Extracellular vesicles
GMP: Good manufacturing practice
hMSCs: Heterogenous parental MSCs
IL-10: Interleukin-10
IUAs: Intrauterine adhesions
Α-SMA: Alpha smooth muscle actin
LIF: Leukemia inhibitory factor
MSC: Mesenchymal stromal cells
TGF-β: Transforming growth factor beta
TNF: Tumor necrosis factor-*α*
VEGF: Vascular endothelial growth factor
