# Supplementary figures and images for: Homogenous subpopulation of human mesenchymal stem cells and their extracellular vesicles restore function of endometrium in an experimental rat model of Asherman syndrome
Source: Stem Cell Res Ther. 2023 Apr 3;14:61. doi: 10.1186/s13287-023-03279-7 (PMC10071639; doi:10.1186/s13287-023-03279-7)

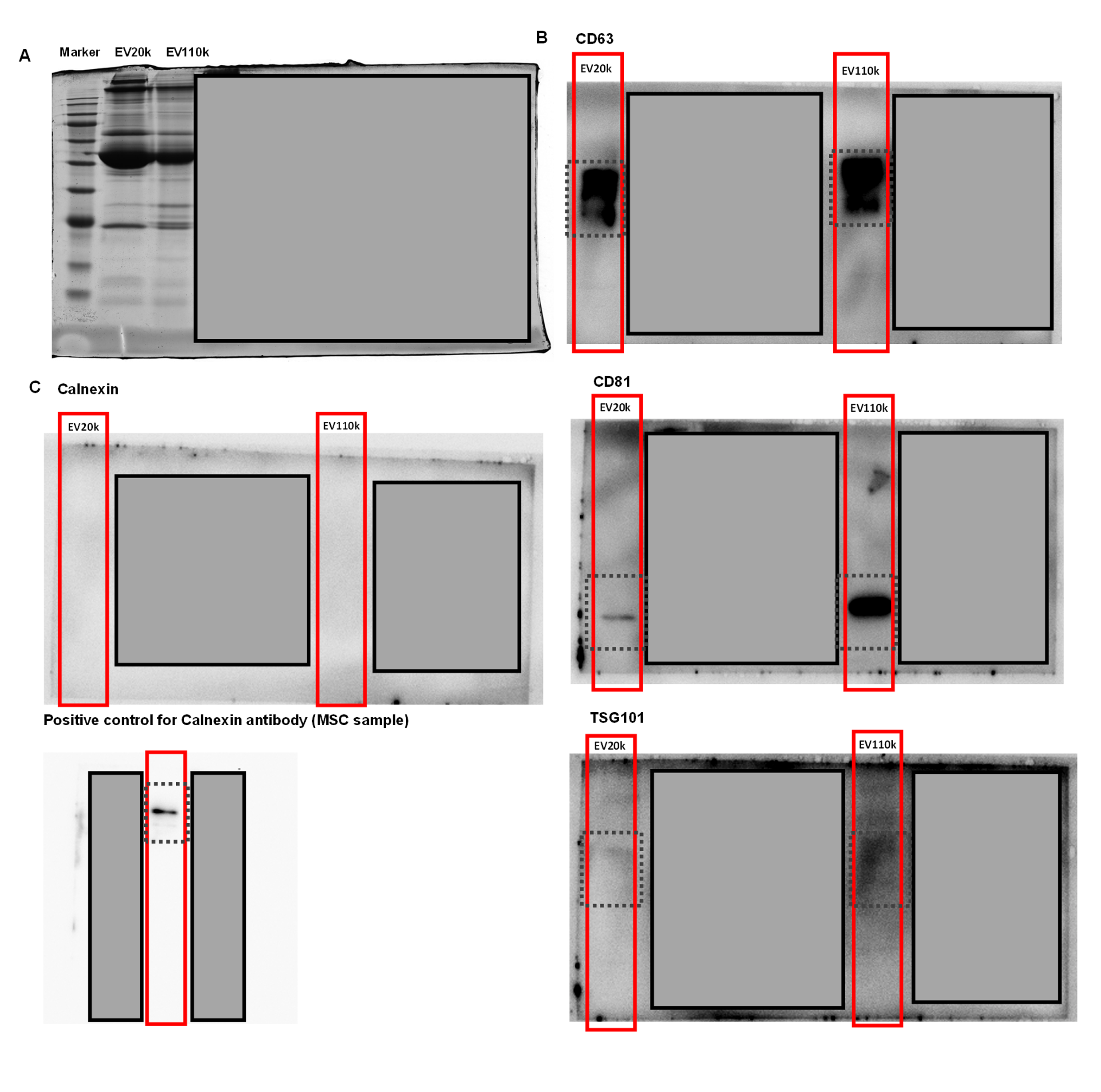

Supplement: Supplementary file 1 — Additional file 1: Fig. S1. Full-length Western blots and SDS-PAGE of proteins extracted from EVs. (A) The protein extracted from EVs was loaded on SDS-PAGE and stained with Coomassie Brilliant Blue (CBB) and confirmed the protein content estimation by BCA. The resulting SDS-PAGE (red box) confirmed distinct protein profile of the EV20k and EV110k. The blue box represented the remain part of the gel that is related to other samples not related to this manuscript. (B) Full-length Western blots of corresponding bands of CD63, CD81, and TSG101. (C) The expression of Calnexin as a negative marker was tested and cMSCs lysate were used as positive control for Calnexin expression. The equal amount of proteins from two samples (EV20k and EV110k) were loaded into the SDS-PAGE and blotted based on detailed protocol in the method session and the blots were detected by antibodies. The resulting blot (red box) represent the whole-body image of blot and the dotted box represent the cropped band for the Figure 1C. The grey box related to other samples blot (not related to this study) that were run in the same gel. [file 13287_2023_3279_MOESM1_ESM.tif]

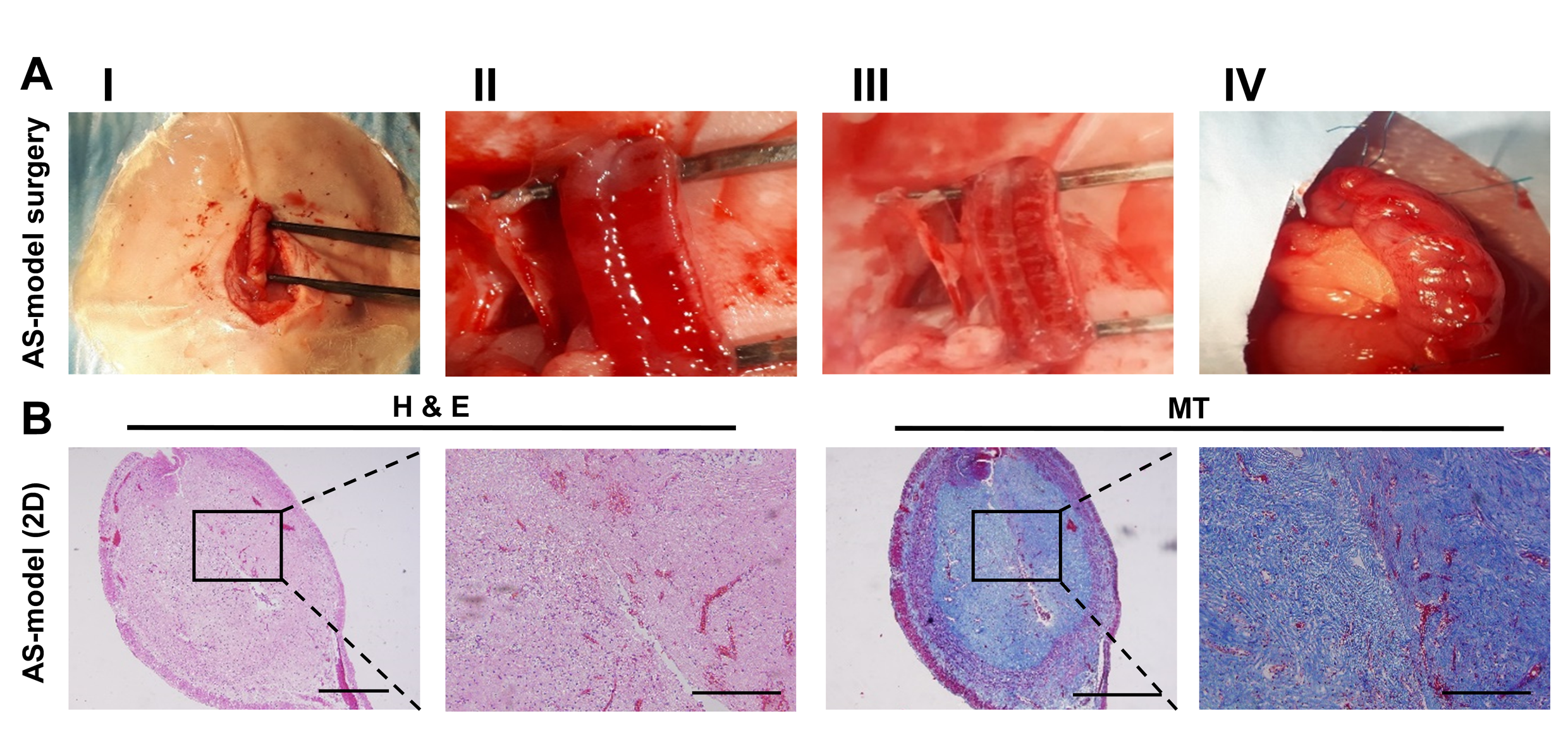

Supplement: Supplementary file 2 — Additional file 2: Fig. S2. Establishment and confirmation of the Asherman syndrome (AS) model in Wistar rats. (A) The surgical procedure. I. A vertical incision (approximately 1.5 cm) was made in the lower abdomen to expose the uterine horns. II. Excision of two-thirds from each uterine horn wall. III. Curettage was performed by scratching the inner uterine surfaces until the uterine walls became rough and pale. IV. Suturing and wound closure. (B) Histopathology of uteri in the normal and the AS model two weeks after surgery to ascertain the amount of endometrial damage. Hematoxylin and eosin (H&E) and Masson’s trichrome (MT) staining. Scale bar in the left side: 1000 µm, scale bar in the right side: 200 µm. [file 13287_2023_3279_MOESM2_ESM.tif]

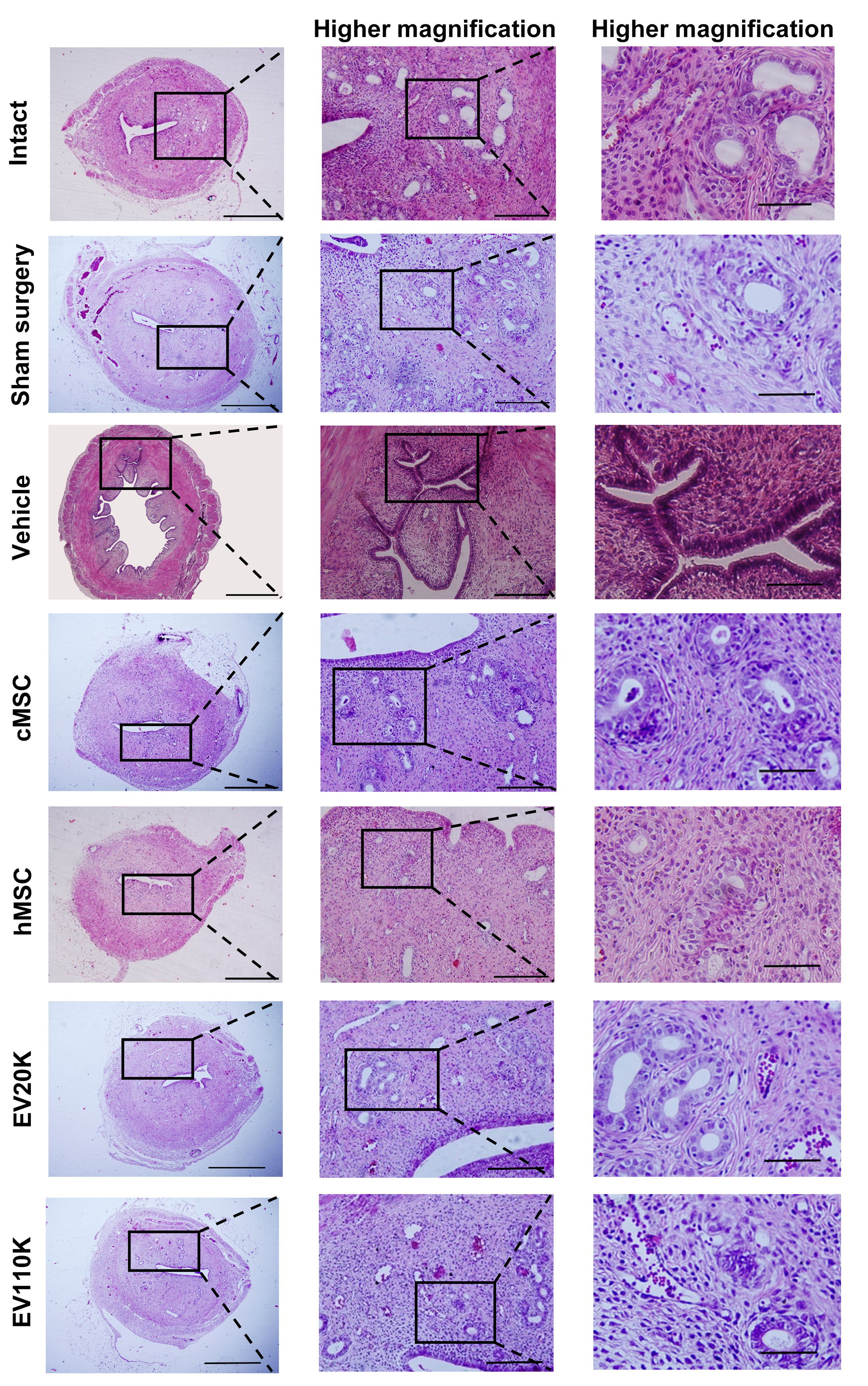

Supplement: Supplementary file 3 — Additional file 3: Fig. S3. Hematoxylin and eosin (H&E). Scale bar in the left side: 1000 µm, scale bar in the right side: 100 µm. [file 13287_2023_3279_MOESM3_ESM.tif]

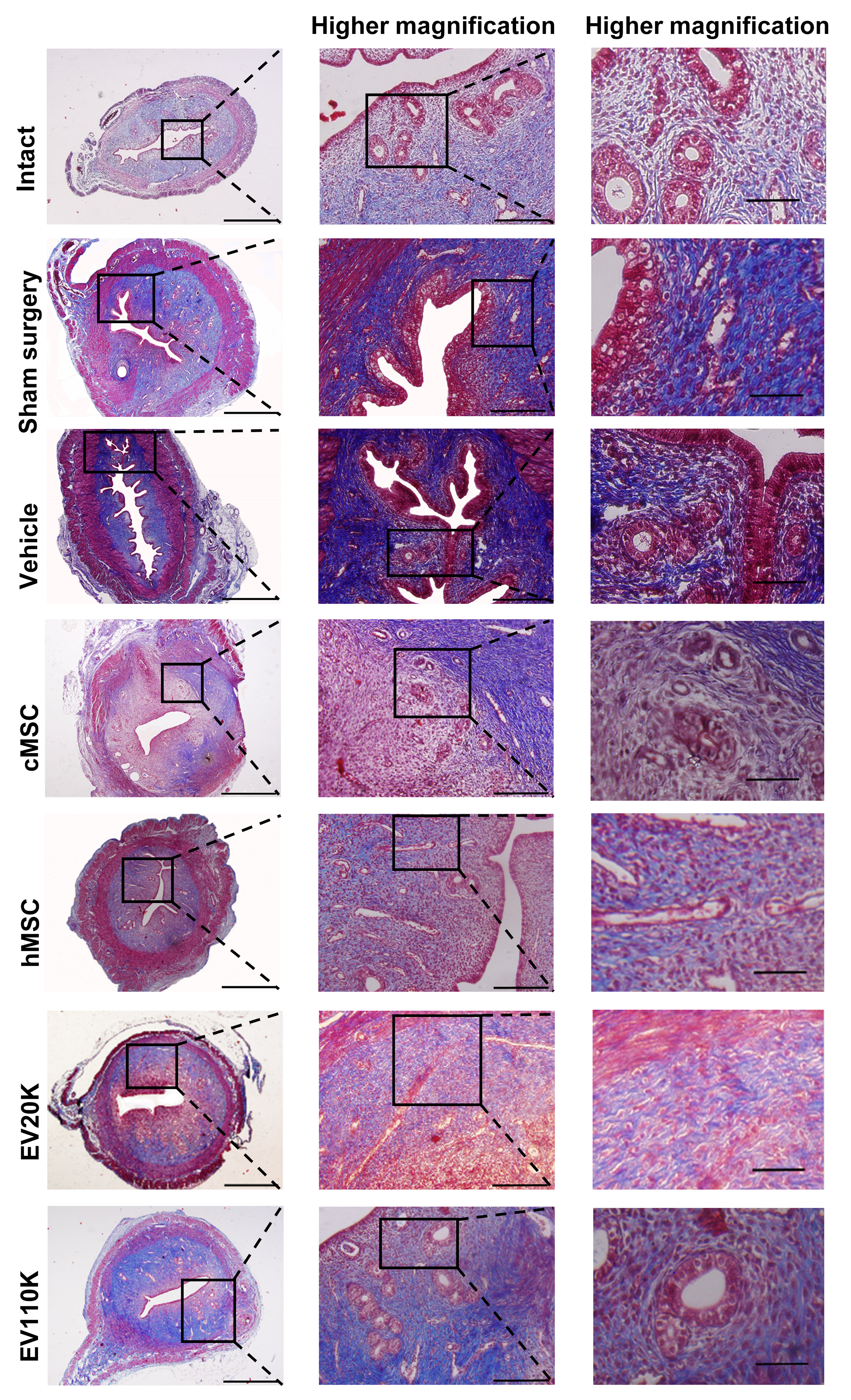

Supplement: Supplementary file 4 — Additional file 4: Fig. S4. Masson’s trichrome (MT) staining. Scale bar in the left side: 1000 µm, scale bar in the right side: 100 µm. [file 13287_2023_3279_MOESM4_ESM.tif]
